# Notes and Notices

**Published:** 1887-06

**Authors:** 


### NOTES AND NOTICES.

Dr. Frederick Bruns has removed to Hotel Pelham, corner Tremont and Boylston Streets, Boston, Mass.

Dr. J. Martine Kershaw held an "at home" to the members of the Missouri Institute of Homœopathy, Tuesday evening, April 26th, at his residence, 3500 Laclede Avenue, St. Louis.

Evils of Quinine.—Hahnemann declares that the evil effects of dosing with Quinine are most difficult to cure; even more so than that following syphilis and mercury. Remember this, before you venture upon using any Quinine.

Logic of Failure.—In "*The Hahnemannian*" *Monthly* for April, the editor states "there *are* logical and scientific reasons" for the employment "in certain cases of other means and measures non-homœopathic." We only know of one reason—failure!

Alumni Association of Hahnemann Medical College.—The annual meeting of this Society was held at new college building on Thursday evening, April 7th. One hundred new members were elected. The present membership is four hundred and forty.

Dr. S. Lilienthal writes to us, under date of April 19th, that the San Francisco College opens in a week "with a good class." The Doctor promises that "the teaching of the *Organon* shall not be neglected, as it is delegated to your humble servant, S. Lilienthal." We wish him success in combating mongrelism.

To Prevent Colds.—In a recent journal some advice as to how to prevent taking cold in traveling was given. After advising as to clothing, etc., this remarkable rule was given: "Every adult should take five or ten grains of Quinine on going to bed (in sleepers)!" How the innocent laity are stuffed with this vile drug!

Poor Hering! The "Dean of the Hahnemann Medical College," of this city, recently told one of his pupils that Hering's *Guiding Symptoms* were of no use! Why then did the worthy Dean act as President of the Society formed solely for the purpose of publishing the very volumes he now declares to be worthless? Rats!

Not Isopathy.—The homœopathic doctrine has never taught to cure a disease by the very same agent which had produced it. This has been repeated to the foolish opponents of Homœopathy over and over again, although to all appearances in vain. Homœopathy professes to cure diseases by means of drugs which produce exactly similar, but not identical, symptoms.—*Hahnemann.*

Only Something in It.—A short while ago our esteemed brethren of the old school proclaimed, amid much rejoicing, that *at last* a real specific for phthisis had been found in the gas bag of Dr. Bergeon. Now "the conclusion so far reached seems to be that while there may be 'something in it,' the treatment is very far from a specific for consumption."—*Medical Record.* Only gas, after all!

Another Cure for Tuberculosis.—Some "celebrated French physicians" have discoverd another cure for tuberculosis! This time it is tannin, a while ago it was carbonic acid gas, to-morrow it will be something else. It is "a cold day" when some "celebrated French physician" does not discover a certain cure for consumption, and yet the dread destroyer marches on unchecked!

Sound Homœopathy.—A true physician will beware of forming a predilection for any particular remedies which chance may sometimes have led him to administer with success. This preference might cause him to reject others which would be still more homœopathic, and consequently of greater efficacy.

He must, likewise, be careful not to entertain a prejudice against those remedies from which he may have experienced some check, because he had made a bad selection, and he should never lose sight of this great truth, that of all known remedies there is but one that merits a preference before all others, viz.: that whose symptoms bear the closest resemblance to the totality of those which characterize the malady. No petty feeling should have any influence in so serious a matter.—*Hahnemann.*

The Diploma Dispute.—Drs. Pease, McNeil, and others have charged the Hahnemann Medical College, of San Francisco, with issuing diplomas to certain persons, who were not proper recipients, nor properly qualified. The charge is a grave one, and now is fully detailed in a pamphlet recently issued. The testimony seems to be against the College. We can only deprecate such doings. Too much of this kind of "graduating" has been done in our colleges. It is simply a disgrace to the profession.

Suppositories of Ice.—According to the *Mass. Med. Journal*, Dr. Chenee in an article in *Jour. de Med. et Chir.* recommends ice suppositories in retention of urine. He introduces a piece of ice the size of a chestnut every two hours. Urethral spasms cease and in the course of two hours urine flows. The ice acts also as a sedative, rendering the introduction of bougies and sounds into the bladder comparatively painless.

A Judge who Knows.—A Mrs. Sarah Patterson was recently prosecuted in this city before Judge Arnold for unlawfully practicing medicine. Her defense was that she was not a doctor but a spiritualistic medium, and that she did not prescribe herself, but merely conveyed prescriptions from the dead to her patients.

She was accused of giving one patient "blood-root and juniper berries dissolved in gin." Judge Arnold asked who was responsible for that prescription? (We quote now from the report:)

Mr. Kilgore.—"I'll tell you. His name is Constantine Hering. He was a prominent homœopathic physician, and has been dead for many years."

"Well," said the Court, "he certainly was not a homœopath if he prescribed blood-root, juniper berries, and Holland gin. You'll have to get another one." This created another laugh.

The Library of the Surgeon General's Office is the most complete Medical Library, with the best subject index in the world. Hitherto physicians have not always been able to obtain reports of cases or other references from this source of information, because they have not known of any one upon whom they could rely to go to the Library, examine the subject catalogues and supplementary indices, find the required references, and make the necessary transcripts. This Bureau has performed these services for physicians and medical authors, and is prepared to attend promptly to any such work that may be ordered. Terms, one dollar an hour per research; ten cents a folio for type-writer transcripts, and thirty cents a folio for translations.

Joseph B. Marvin, *Manager.*

*The Bureau of General Information, office in the Corcoran Building (opposite the Treasury), P. O. Lock Box* 379, *Washington, D. C.*

Seceders Rewarded!—In connection with Dr. Lippe's paper upon the Women's Homœopathic Association of Pennsylvania, it will be of interest to note the report that the seven seceding physicians have been promised positions in the hospital that is to be built in connection with the (so-called) "Hahnemann Medical College of this city. They can use there all the crude drugs, opiates, etc." their best judgment may fancy.

